# Multi-Context Strategies and Opportunities for Increasing Levels of Physical Activity in Children and Young People: A Literature Review

**DOI:** 10.3390/children11121475

**Published:** 2024-11-30

**Authors:** Víctor Arufe-Giráldez, Javier Pereira Loureiro, María Betania Groba González, Laura Nieto Riveiro, Nereida María Canosa Domínguez, María del Carmen Miranda-Duro, Patricia Concheiro Moscoso, Rocío Rodríguez-Padín, Javier Roibal Pravio, Manuel Lagos Rodríguez, Oliver Ramos-Álvarez

**Affiliations:** 1 Research Group in Technology Applied to Occupational, Equality and Health Research, Faculty of Education, University of A Coruña, 15008 A Coruña, Spain; rocio.rodriguez.padin@udc.es; 2 Research Group in Technology Applied to Occupational, Equality and Health Research, CITIC Research Center, University of A Coruña, 15008 A Coruña, Spain; javier.pereira@udc.es (J.P.L.); b.groba@udc.es (M.B.G.G.); laura.nieto@udc.es (L.N.R.); nereida.canosa@udc.es (N.M.C.D.); carmen.miranda@udc.es (M.d.C.M.-D.); patricia.concheiro@udc.es (P.C.M.); javier.roibal@udc.es (J.R.P.); m.lagos@udc.es (M.L.R.); 3 Faculty of Education, University of Cantabria, 39005 Santander, Spain; oliver.ramos@unican.es; 4 Spain and Applied Technology Research Group for Research in Employment, Equality and Health, University of A Coruña, 15008 A Coruña, Spain

**Keywords:** physical activity, infancy, physical exercise, childhood, adolescence, health

## Abstract

Background: In today’s society, low levels of physical activity are observed in the child and adolescent population, which can cause numerous pathologies, such as obesity and mental health problems. Objective: This article aims to compile all the contexts and scenarios where it is possible to increase the levels of daily physical activity of children and young people, and which have significant scientific support. Method. To do so, a literature review was carried out examining four key contexts for intervention: school, extracurricular, family, and socio-community. Results: The results indicate that the school context, with strategies such as physical education classes and active breaks, is crucial but insufficient on its own, so it is essential to complement it with interventions in extracurricular, family, and socio-community environments. The involvement of families, access to adequate infrastructure such as parks and green areas, and the responsible use of technology, including active video games and the role of influencers on social networks, are presented as key elements to combat a sedentary lifestyle. Conclusions: It is important to highlight the importance of establishing socio-educational programs that adopt a comprehensive approach to promote physical activity in children and youth, highlighting the scientific evidence that supports the effectiveness of intervening in multiple scenarios. This review concludes that a coordinated approach between different actors (schools, families, communities) is necessary to ensure that children and youth reach adequate levels of physical activity, which not only improves their physical health, but also their mental well-being and cognitive development.

## 1. Introduction

In recent decades, the child and youth population has become a vulnerable population to suffer from numerous pathologies and diseases associated with a sedentary lifestyle [1,2,3,4,5,6,7,8]. Today’s society has certain characteristics that invite all individuals to adopt a sedentary lifestyle, with the consequent risk to their health [7]. Some of these characteristics are as follows: the technological abundance of screen devices in homes, registering a high number of devices per home and the excessive use of these [9,10,11,12,13,14,15], a poor diet and a wide range of ultra-processed foods and fast food [16,17,18,19,20], or the lack of time and adequate spaces to do physical exercise in cities, among other characteristics [21,22,23]. One of the problems that a sedentary lifestyle brings in today’s society is the high prevalence of overweight and obesity in infancy and childhood recorded in the last two decades [24,25,26,27].

It has been scientifically proven that physical activity (PA) acts with a double function on the health of the population [28,29,30]. The preventive function, helping to avoid a large number of pathologies, including cardiovascular, respiratory, osteoarticular, and muscular problems, psychological problems as well as other pathologies such as Parkinson’s [31], diabetes [32], and digestive problems [33], among other pathologies. And the rehabilitation function or improvement in the quality of life of people diagnosed with multiple pathologies. On the other hand, various studies confirm that the main causes of death in both the youth and adult population are associated with environmental factors, mainly lifestyle [34,35]. Therefore, many of these diseases could be prevented or their onset delayed through programs to promote the practice of PA, since an active lifestyle with adequate levels of PA is associated with a lower development of cardiovascular, respiratory, and neurological pathologies, among others. Even if there is a genetic predisposition to suffer from a cardiovascular disease, its onset can be delayed or avoided. However, a bad lifestyle, whether or not there is a genetic predisposition to contract a cardiovascular pathology, is always associated with a higher risk [36]. PA is understood as the performance of any bodily movement that involves a metabolic expenditure greater than the basal one, and physical exercise is the planning and programming in space and time of PA for the purpose of improving health and different physical capacities [37]. Any amount of PA can have a positive impact on people’s health [38]. For all these reasons, it is important to establish the greatest number of strategies that promote the practice of PA in the child–youth population in all contexts where this under 18 years of age population operates. Some authors [35] confirm that with only 1 h of daily PA carried out in childhood and adolescence, the probability of suffering from mental health problems in adulthood is reduced, without forgetting that more than 20% of minors present some type of mental pathology in childhood due to a sedentary lifestyle [10,39]. Thus, the combination of lower amounts of PA and more time using screen devices leads to a higher prevalence of mental health problems in childhood [40], and on the other hand, less screen time and a PA count greater than 12,000 steps are associated with better cardiovascular health in adolescence [41].

It is also important to note the recommendations of the World Health Organization (WHO) [42] which emphasize the need to ensure at least 180 min of PA daily in early childhood. However, a recent study [43] carried out on a sample of more than 7000 children from 33 different countries found that, from the age of 3 to 4, 84% of this population does not comply with the recommendations for sleep, blood pressure, and hours of screen time recommended by the WHO, which invites reflection and concern about the health of infancy and childhood. In adolescents the data are similar, highlighting the latest studies [44] that more than 80% of adolescents do not meet the WHO PA recommendations.

The purpose of this paper is to review the scientific literature on the many possible contexts where children and adolescents can increase their PA levels, thus bringing together in a single reference document all the possible strategies for promoting PA in infancy, childhood, and adolescence in different contexts that have been scientifically endorsed. It aims to position itself as a reference article for researchers and political and educational leaders in the field of PA and health in infancy, childhood, and adolescence, given its integrative nature of all the possible contexts where political–educational–social authorities and families must act to promote the increase in physical activity levels in minors.

## 2. Materials and Methods

For this work, it was decided to carry out a literature review given the topic addressed and its extension, which makes it impossible to carry out a systematic review or a meta-analysis. The literature review is a research method based on the search for relevant information on a given topic, subsequently carrying out an exhaustive and critical analysis of the selected sources [45]. This involves a systematic examination of academic sources to provide an overview of the important literature in the research area.

### 2.1. Procedure for Searching and Selecting Articles

For this work, the Web of Science, Scopus, and Dimensions databases were used to search for scientific documents, given their high volume of indexed scientific works. To search for documents, the following descriptors were used in the title, abstract, and keywords fields: [childhood AND physical activity]; [childhood AND physical activity]; [physical education AND physical activity]; [center programs AND physical activity]; [active breaks AND physical activity]; [motor wedges AND physical activity]; [active playgrounds AND physical activity]; [green areas AND physical activity]; [extracurricular activities AND physical activity]; [play in the street AND physical activity]; [active transport AND physical activity]; [physical activity AND family]; [active families AND physical activity]; [free play at home AND physical activity]; [active toys AND physical activity]; [active video games AND physical activity]; [wearables AND physical activity]; [active cities AND physical activity]; [influencers AND physical activity]; [social media AND physical activity]; [playgrounds AND physical activity]; [green cities AND physical activity]; [advertising AND physical activity]. The search was carried out between January and October 2024. All researchers participated in both the search and the selection of information sources, subsequently holding a debate and consensus on which scientific documents to include in the review. Each context was assigned to two researchers, for a total of 8 researchers and 4 contexts. The other researchers provided support in the process of selecting and supervising the leaked documents.

### 2.2. Inclusion and Exclusion Criteria

The following inclusion criteria were applied: (1) include only scientific articles; (2) published in English or Spanish; (3) give priority to systematic review articles, meta-analysis or other types of reviews; (4) in the event of not finding articles that met criterion 3, the option was to search for articles with an experimental or quasi-experimental design; (5) articles with a solid methodology and large population samples.

### 2.3. Data Synthesis and Explanation of the Scenarios or Contexts

For the synthesis of the data, it was decided to work with a maximum of 5 articles in each scenario to avoid writing a very long article. With the information from these works, the results and discussion section was carried out in each of the established scenarios.

A subtotal of 21 scenarios assigned to 4 macro contexts were established: school (to refer to those actions carried out within the school environment), extracurricular (referring to actions carried out outside school hours at the educational center), family (referring to actions carried out within the family), and socio-community (referring to actions promoted in cities).

### 2.4. Explanation of the Scenarios or Contexts

**Figure 1:**
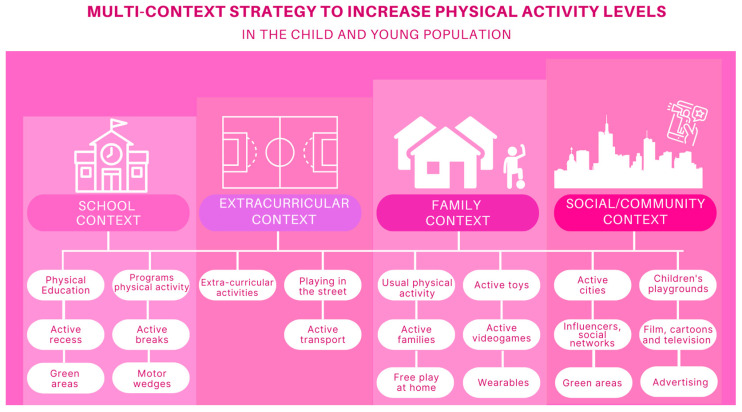
Multi-context strategy to increase physical activity levels in the child and youth population.

The study proposes a multi-scenario approach focused on four main contexts (Figure 1): the school environment, the extracurricular environment, the family environment, and the socio-community environment. Each of these contexts offers unique opportunities and strategies endorsed by the scientific community to promote PA in the child–youth population.

In the school environment, physical education (PE) programs, active recess, motor wedges, active breaks, green recreation areas, and comprehensive projects that promote movement during school hours will be analyzed. Interventions based on scientific evidence that have proven effective in increasing children’s PA in the context of being attached to educational centers will be evaluated.

In the extracurricular environment, organized sports activities, sports clubs, and free-time programs that encourage children’s participation outside of school hours will be reviewed. The importance of playing in the street and active transportation will also be analyzed. This review will consider studies that have evaluated the effectiveness of these programs in different cultural and socioeconomic contexts.

The family environment will be analyzed considering the family dynamics and practices that influence children’s PA levels. Interventions aimed at parents and caregivers that promote active lifestyles at home, parental lifestyle, active play at home, active video games, active toys, and the use of wearable technologies, among others, will be explored.

Finally, the socio-community environment will be investigated in terms of the available infrastructure, parks and public spaces (active cities), and community programs that facilitate access to and participation in physical activities. Open spaces in cities are areas that provide opportunities for children to engage in PA, which is crucial for their health and development, and they do not have to be green areas or natural spaces. These spaces include open green spaces, public parks, and open areas in the neighborhood, which are designed or naturally available for recreational and physical activities.

The role of influencers, famous personalities and athletes, and their influence on young people, advertising, film, television, and cartoons to promote sports practice will also be investigated, in addition to analyzing the role of playgrounds, multi-adventure parks, etc. This review will include studies that examine how features of the built environment and local policies may influence children’s active behaviors.

### 2.5. Ethical Considerations

In this literature review study, the ethical recommendations indicated by Paz Maldonado [46] were followed. As it is a documentary work, compliance with the ethical principles relevant to this type of research were guaranteed. In particular, academic integrity was taken care of, ensuring an exhaustive and respectful review of the sources consulted. All references used were cited appropriately, recognizing the authorship of the original works and avoiding any form of plagiarism. In addition, an attempt was made to interpret the findings in an objective and transparent manner, without distorting the information in order to favor preconceived ideas. Likewise, the principle of the ethical use of information was considered, presenting the results of the review with the aim of providing relevant and useful knowledge for the educational community, and avoiding applications that may perpetuate inequalities or misinterpretations.

In summary, academic rigor, transparency in the management of information, and respect for the contributions of the authors reviewed were prioritized.

## 3. Results and Discussion

Below are the results and discussion carried out in each of the macro contexts and sub-scenarios.

### 3.1. School Context

#### 3.1.1. Physical Education Classes

PE classes established by law in the educational curricula of many countries constitute an excellent opportunity to improve PA levels in infancy, childhood, and adolescence. Various studies have addressed the benefits of these classes [47]. However, some authors continue to point out that current legislation gives little prominence to this subject within the school curriculum, which means that there are not enough hours to meet the minimum requirements established by the recommendations of international institutions such as the WHO. Some studies [48] even note only one weekly PE session in Early Childhood Education. The enrichment of PE classes with specific physical literacy programs improved the benefits of this subject, confirming, in addition to the PA levels, improvements in the psychological domain (affective and cognitive) and motor competence [49]. Another study [50] showed that increasing organized PA at school from 4 to 10 h of PE per week resulted in improved blood pressure among young adolescents, compared to standard PA levels. Children in the increased PA group showed a higher percentage of very good fitness scores and a lower percentage of poor scores, indicating that higher PA levels positively influence children’s fitness outcomes. Finally, the results of a systematic review of the study were noteworthy: [51] of a total of 39 articles that addressed PE classes, gender differences were observed in the impact of PE on daily moderate-to-vigorous physical activity (MVPA) and general daily PA, which invites the need to adapt physical education classes to the context of each child.

#### 3.1.2. School Physical Activity Programs

The school environment is presented as a setting in which different PA programs can be implemented within the school. There is sufficient evidence to suggest that the promotion of PA through these school programs offers greater opportunities to increase the levels of this [52]. In the scientific literature, there are numerous articles that refer to comprehensive school physical activity programs (CSPAP), these being programs that coordinate PA opportunities for school-aged children through PE, programs offered before and after school and during the school day, and those facilitated through staff participation and community participation [53,54,55]. Some authors [56] point out that to maximize the effectiveness of PA programs within the school environment, it is necessary to improve professional development for teachers, ensuring that PE programs are not only contextually relevant, but also optimized for maximum impact in diverse educational environments.

#### 3.1.3. Active Recess

Recess is another of the settings where PA can be promoted through programs such as those called active recess. Within this context, active schoolyards—spaces designed or adapted to encourage play and movement—have become a key tool for increasing daily PA among students [57]. In a systematic review study [58], the effectiveness of these playgrounds in increasing these levels was analyzed, and the authors concluded that by using strategies such as increased space, color markings, equipment provisions, structured activities and teacher involvement, active recess appears to be effective in increasing PA. Several studies have shown that the implementation of active playgrounds significantly increases the time spent in moderate- to vigorous-intensity activities during recess, compared to traditional playgrounds [59]. A systematic review [60] that analyzed nine articles showed that five studies demonstrated a positive effect of the intervention on children’s PA levels, four reported statistically significant increases, and two reported significant decreases in PA at recess. The authors highlighted the complexity of comparing the studies given their methodological diversity.

#### 3.1.4. Active Breaks

Active breaks, or short periods of PA integrated into the school day, have been shown to offer numerous benefits for increasing PA levels in children and adolescents. These breaks not only help to meet PA guidelines, but also contribute to improved classroom behavior, cognitive function, and overall well-being. A systematic review [61] indicated that such interventions positively influenced classroom behavior and quality of life, also reporting an increase in PA levels in schools. Another study [62] conducted in primary schools in northern New South Wales showed that children who participated in active breaks performed significantly higher MVPA compared to children in control groups, contributing to more students meeting PA guidelines. The BRAVE study [63] highlighted that active breaks are a feasible strategy to incorporate PA into the school day without interrupting academic activities, thus helping students achieve the 60 min of daily PA recommended by the WHO.

#### 3.1.5. Motor Wedges

Motor wedges and increased PA are interconnected concepts that can significantly impact motor skill development and overall health. Motor wedges, as a tool in PE, can facilitate the improvement of motor skills and physical activity levels in childhood and adolescence [64]. On the other hand, the implementation of cooperative motor wedges in the classroom improves the motivational climate of the classroom and enhances the work of the executive functions of the students [65]. Motor wedges are a concept that has been little addressed in the scientific literature, but the studies found indicate that they are another possibility to increase PA levels.

#### 3.1.6. Green Areas or School Forests

Integrating green areas or school forests into school environments has been shown to significantly improve PA among students. These natural environments provide unique opportunities for children to engage in various forms of PA, which can contribute to their overall health and well-being. Some authors [66] have found that playgrounds with green areas promote higher levels of PA, due to the shade of trees among other reasons. A lower density of schoolchildren also favors this. A study [67] in Norway found that both constructed playgrounds and natural forests contributed equally to children engaging in moderate-to-vigorous physical activity (MVPA), with each environment offering unique opportunities for PA. Similarly, forest school programs have been shown to double levels of physically active play compared to typical daycares, highlighting the potential of natural environments to promote vigorous play [68]. Another study [69] found that nearly one-third of children engaged in light, moderate, or vigorous PA, with a notable decrease in sedentary behavior over time. Observations indicated that children were more active in these natural spaces, and follow-up analyses revealed a significant increase in overall PA. The presence of green spaces not only encourages physical participation, but also fosters positive social interactions, which improves overall developmental outcomes for young people.

### 3.2. Extracurricular Context

#### 3.2.1. Extracurricular Activities

The extracurricular environment, beyond school hours, is another setting where children can increase their levels of PA. Extracurricular sports activities play an important role in improving PA levels among children and adolescents [70]. Systematic reviews and studies have explored several dimensions of these activities, including their effectiveness, their impact on psychosocial and health outcomes, and the factors that influence their success. A systematic review and meta-analysis [71] examined the effectiveness of after-school interventions to increase MVPA levels in children and adolescents. After-school interventions produced a modest increase of 4.84 min per day of MVPA, and significant benefits were observed in overweight boys and girls. Another review [72] highlighted that while youth sports participation is positively associated with increased PA levels, the relationship with obesity remains inconclusive. Other work [73] suggests that a comprehensive physical literacy program during the after-school period may be feasible to implement and may lead to improvements in the affective domain of children’s physical literacy, as well as increasing the amount of PA performed. It concludes that while extracurricular sports activities have demonstrated potential to increase PA levels and improve certain health outcomes, their effectiveness is not uniform across demographic groups. Factors such as gender, baseline activity levels, and program design significantly influence outcomes [71].

#### 3.2.2. Play in the Street

Street play interventions, particularly through the implementation of Play Streets, have been explored as a means of increasing PA among children. These interventions involve temporarily closing streets to traffic and creating safe spaces for children to actively play. A systematic review [74] indicated that Play Streets significantly improves opportunities for children and adolescents to engage in PA. Evidence from the reviewed studies suggested that Play Streets lead to increased levels of MVPA, reduced sedentary behavior, and improved community interactions. In another study [75] conducted in Ghent, Belgium, Play Streets were also found to significantly increase children’s MVPA levels and reduce sedentary time. Children living in Play Streets areas increased their MVPA from 27 to 36 min during the intervention, while sedentary time decreased from 146 to 138 min. In rural settings, Play Streets have been successfully implemented, and social influences, such as the presence of other active children, significantly increased the likelihood that children would be active [76]. It is concluded that creating a safe play space near urban children’s homes through the Play Street intervention is effective in increasing children’s MVPA and reducing their sedentary time. Future research should focus on addressing some gaps in published studies, such as how to implement the programs and their evaluation to maximize the potential benefits of street play-related interventions [77]. Furthermore, exploring the integration of Play Streets with other community-based interventions could further enhance its impact on children’s PA and health outcomes. Ultimately, higher quality informal play spaces close to home may help mitigate the decline in children’s MVPA during middle childhood by promoting unstructured active play [78].

#### 3.2.3. Active Transport

Active transport, including walking, cycling, and other non-motorized modes of transport, is increasingly recognized as a vital component of PA that can contribute to public health for children and adolescents. Systematic reviews have explored several dimensions of active transport, including its impact on health outcomes, the effectiveness of interventions to promote it, and its role in different populations. A recent systematic review [79] noted that active transport generally results in net additional PA without displacing other forms of PA. Most of the studies reviewed confirmed that active transport contributes to overall PA levels, with minimal compensatory reduction in other domains. In Europe, active transport to school has been identified as a strategy to increase PA among children and adolescents, although the efficacy of interventions varies [80]. Active transportation use varies across demographic groups, with disparities in access to infrastructure. Underrepresented populations, including minorities and low-income groups, often face barriers to accessing active transportation, such as poor accessibility to bicycle infrastructure [81]. While active transport offers significant health benefits and can increase overall PA levels, challenges remain to promote its widespread adoption. Addressing infrastructure, safety, and accessibility issues is crucial, particularly for underrepresented populations. Furthermore, understanding the diverse perceptions and needs of different demographic groups can improve the effectiveness of interventions aimed at increasing active transport. Future research should focus on developing comprehensive strategies that integrate environmental, social, and behavioral components to maximize the health benefits of active transport [82].

### 3.3. Family Context

#### 3.3.1. Regular Physical Activity

Regular PA in children is crucial for their physical, mental, and cognitive development. Research highlights the multifaceted benefits of increased PA and the importance of structured interventions to promote active lifestyles among children. A literature review study [83] highlighted the significant benefits of regular PA in adolescents, particularly for improving lipid profiles and cardiovascular health. Another systematic review and meta-analysis [84] indicated that regular exercise can improve lung function parameters, specifically forced vital capacity and forced expiratory volume, in healthy children and adolescents. The review highlighted the need for further well-designed studies due to the limited number of existing studies and significant heterogeneity among them, emphasizing the importance of this public health issue. Another systematic review and meta-analysis study [85] found that regular PA interventions significantly improve children’s perceptual abilities, particularly visual perception and executive functioning.

#### 3.3.2. Active Families

Active family involvement plays a crucial role in improving children’s PA levels. Research indicates that family-based interventions can positively influence children’s PA behaviors, although effectiveness varies depending on intervention design and family dynamics. Parents are role models for their children, and their PA behaviors are positively associated with their children’s PA behaviors. In this systematic review [86], the findings highlight that while parental PA may influence children’s activity levels, the effect is modest and consistent across child age groups and parent–child gender combinations. Studies have shown that there is a consistent association between parental and child emotional attitudes, with stronger correlations observed in same-sex dyads (e.g., father–son, mother–daughter). Parental involvement in organized physical activities is linked to higher levels of children’s participation in extracurricular sports activities. Thus, boys with active fathers and girls with active mothers, particularly those who participate in organized physical activities, tend to play more sports and more frequently [87]. Family-based interventions have been shown to effectively increase children’s PA levels. A meta-analysis found that such interventions have a small but significant effect on increasing PA, especially for daily measures [88]. In another systematic review [89] findings suggested that fostering an active family environment can improve children’s PA levels, especially daughters. Due to current living conditions, many parents are forced to leave their children with grandparents. Some authors [90] have studied this environment and analyzed how much PA children do when they are cared for by their grandparents. These studies highlighted that grandparents cared for their grandchildren on average 12 h per week, and in most cases, a low level of PA was observed in the children, without meeting the WHO recommendations for PA, and only 6% of the children played in the street. These findings highlight the importance of training grandparents to make them aware of the need to go for walks with their children or play in the street or in playgrounds; benefits of PA that will also be obtained by the grandparents themselves.

#### 3.3.3. Free Play at Home

Free play at home is a critical component of increasing children’s PA levels as it offers a flexible and engaging way for children to be active. Free play in children refers to unstructured and self-directed play that allows children to make their own decisions about what, when, and how to play. It is a crucial component of child development, as it promotes physical, mental, and social health [91].

Research [92] indicates that physical environmental conditions promote active play and have a positive effect on PA in preschoolers. A study [93] found that children’s active free play in their home yard is inversely associated with preferences for non-physical activities, suggesting that children who play more freely at home may have higher PA levels. It is important to highlight the findings of some studies [94] that showed that active play at home will depend on the equipment that the child has in his or her home. The presence of active play equipment, such as trampolines and bicycles, is associated with higher levels of PA. Conversely, the abundance of electronic devices may reduce the number of days that children meet PA guidelines. In some contexts, such as in the case of Chinese children, the availability of sports equipment at home is positively associated with MVPA [95], so equipping the home with sports equipment will invite children to be more active.

#### 3.3.4. Active Toys

The term active toys refers to those toys that invite the child to move; for example, a bicycle, a stick with a wheel at the end, a ball, the famous twister game...they are toys that are not meant to be sat on, but rather to be moved. Active toys are crucial for children’s development, offering opportunities to improve physical skills, coordination, and overall health [96]. Research indicates that active toys and play interventions can significantly impact children’s PA levels and fundamental motor skills (FMS). The results of a study [97] conducted with a sample of children from the United Kingdom suggest that a relatively short session of unstructured active play with toys or toy substitutes can make an important contribution to a child’s daily level of PA. Another study [98] that analyzed the impact of a socially assisted robot (SAR) found that it offers advantages through rewards that cause children to interact with the SAR by staying up longer during free play.

#### 3.3.5. Active Video Games

Video games, particularly active video games (AVGs) and exercise games, are increasingly recognized for their potential to promote PA among children. These games require physical movement to play, so they offer a novel way to get children to exercise. Research indicates that AVGs can have a positive impact on children’s physical health, motor skills, and weight management, although effectiveness may vary depending on the type of game and the context in which it is used [99,100]. A meta-analysis study [101] found that AVGs are effective in achieving vigorous, moderate–vigorous, and moderate levels of PA, and in reducing BMI and body fat among children and adolescents. Dance appears to be the best option for reducing BMI among AVG subcategories. Another recent systematic review [102] confirmed that home-based interventions, particularly those incorporating active video games and telehealth support, have shown some success in increasing children’s PA. These interventions typically focus on fostering social support and self-efficacy, which are crucial for maintaining PA habits.

#### 3.3.6. Wearable Technology

Wearable devices have emerged as a promising tool for promoting physical activity among children, offering both motivational and monitoring benefits. Wearable devices are electronic devices that can be worn on the body, often in the form of watches or straps, designed to monitor various health indicators and promote PA [103]. These devices, such as the Garmin Vivofit Jr. 3 and the Fitbit Ace 3, are designed to estimate MVPA in children, although their accuracy in free-living conditions remains a concern. Studies have shown that while these devices may overestimate MVPA, they still have potential to encourage increased activity levels through engaging interfaces and real-time feedback [104]. Thus, some authors who have conducted systematic reviews have shown that portable devices positively influence daily MVPA levels and reduce sedentary behavior in children and adolescents [105]. These types of interventions using wearable devices have also been shown to have significant effects on obesity-related outcomes, such as reduction in BMI and body fat, highlighting their role in obesity prevention and treatment [106]. Finally, it is worth noting that one study [107] found that a large majority of school staff had never used wearable devices for teaching or support, but those who did use them primarily used them during physical education or during the school day to monitor and improve students’ PA levels. Most staff expressed a willingness to incorporate wearable devices in the future to promote and educate students about PA, indicating that there is potential for broader implementation in school-based interventions.

### 3.4. Socio-Community Context

#### 3.4.1. Active Cities

The creation of active cities has been a concern for the last decade. Active cities play a crucial role in promoting PA, which aligns with several Sustainable Development Goals (SDGs), in particular SDG 3 (good health and well-being) and SDG 11 (sustainable cities and communities). Integrating physical activity into urban planning and community initiatives can significantly contribute to achieving these goals [108].

Political authorities must give priority to public health and the promotion of PA by creating so-called active cities. Some strategies that these types of cities have in common are increasing the levels of PA in the population, promoting active transport, creating greenways and parks, and organizing sports events, among other strategies [109]. A study [110] systematically reviewed and evaluated the evidence on the physical health benefits of participation in various recreational sports. A total of 136 articles from 76 studies with 2.6 million participants were included. The results show that cycling, football, handball, running, or swimming reduce mortality and decrease the risk of suffering from multiple pathologies. The authors conclude that participation in recreational sports such as these is associated with multiple physical health benefits.

#### 3.4.2. Influencers and Social Media

Influencers have become instrumental in promoting PA in young people through social media platforms, particularly Instagram, by leveraging their reach and credibility to motivate audiences to adopt healthier lifestyles. An influencer is a person who has established a significant presence on social media platforms, allowing them to influence the opinions and behaviors of their followers. These people often share personal experiences and knowledge, creating an emotional connection with their audience [111].

These influencers often offer exercise routines, nutritional advice, and personalized training programs, which can significantly impact the PA levels and lifestyle choices of their followers. The engagement strategies employed by influencers, such as visual content and digital marketing techniques, are crucial to building trust and encouraging active participation among their audience [112]. A study in the United States found that Instagram users who follow fitness influencers are more likely to meet the World Health Organization’s recommendations for moderate PA. This group is predominantly made up of women and millennials who spend a lot of time on Instagram looking for information about exercise and nutrition [113]. Social media interventions, which use peer influence, have demonstrated potential to promote PA among adolescents. These interventions typically involve training social media influencers to encourage PA among their peers [114]. Several studies have explored the impact of social network interventions (SNI) on children’s physical activity, highlighting the potential of these networks to encourage healthier lifestyles. These interventions often leverage peer influence and social connections to encourage more active behaviors, with schools being the primary setting for such initiatives. The effectiveness of these interventions is often linked to the strategic selection of influencers and the integration of social network analysis techniques. Social network interventions are often based on theories such as the self-determination theory and use social influence as a mechanism to promote PA [115]. While influencers and social media play an important role in promoting PA, the effectiveness of their influence can vary depending on demographic factors, the credibility of the influencer, and the dynamics of social media [116]. Furthermore, integrating social marketing strategies and media literacy can further enhance the impact of influencers in promoting PA.

#### 3.4.3. Green Areas

Socio-political authorities can promote the use of green areas to increase population PA levels. Green areas play an important role in promoting PA among children, as they provide natural environments that encourage movement and play. The availability and quality of green spaces can influence the level of PA, which is crucial for children’s physical and mental well-being. Improving access to green spaces can positively influence youth PA patterns, aligning with broader initiatives to improve community health through increased availability of parks [117]. Studies have shown that green spaces have different impacts on children’s activity levels, depending on factors such as urbanicity, socio-demographic characteristics, and the design of the green spaces themselves. One option is the creation of green spaces, such as greenways and parks, within the built environment. Investment in these spaces is an intervention that has the potential to increase physical activity levels among both children and adults. These green spaces can provide an attractive environment, easy accessibility, opportunities for social interaction, stress reduction, and essential services and infrastructure. The results of a systematic review study with meta-analysis [118] to evaluate the effectiveness of greenway interventions in promoting PA found that greenways are effective in promoting PA, with improvements in active travel and MVPA. In addition, greenway characteristics and the duration of exposure were found to influence PA levels. It is concluded that greenway construction is an effective public health intervention to increase PA. Another study [119] indicated that the presence of green areas in urban parks contributes significantly to increasing PA levels among children. Specifically, open green spaces, diverse vegetation, and the availability of trails encourage children to engage in vigorous activities. Related to another setting, namely school playgrounds, a review study [120] indicated that greening school playgrounds has mostly positive effects on social and mental health, but the relationship with increased PA is mixed. While some interventions, such as making playgrounds, have been shown to improve PA levels, there is no consistent evidence that greening specifically leads to increased PA in children. Therefore, while greening may benefit social and mental health, its impact on PA requires further research to establish clear results.

#### 3.4.4. Children’s Playgrounds

Playgrounds have also been the subject of research in the field of PA. The typology of these can determine higher levels of PA in children or even the dominance of certain motor skills over others. Thus, some authors [121] have studied children’s participation in three types of playgrounds: traditional, modern, and multi-adventure. They concluded that all have the potential to contribute to daily PA needs, but a lower amount was recorded in the multi-adventure park and a higher amount in the traditional park. A total of 38% of the time was spent on MVPA. The most observed skills were walking, running, and climbing. Political authorities should create larger playgrounds to increase the distance of movement in the park, thus favoring the time of PA, while combining different structures that promote the work of various motor skills, such as throwing, jumping, turning, hanging, etc. In another study [93], positive correlates in more frequent park/playground play on weekdays were found including the family going to the park together weekly on weekdays and weekends.

Another systematic review study [122] that analyzed investment in outdoor parks and its relationship with PA levels concluded that there is an impact of renovations and the creation of new parks on increasing PA; 26 studies were included, obtained from a search in PubMed, Scopus, and Web of Science in March 2022. Of the studies on park renovations, 65% showed a positive effect on PA, while 35% reported no improvement. Regarding new facilities, 56% observed an increase in PA. These results underline the importance of considering the context and needs of the community before implementing interventions in parks.

#### 3.4.5. Film, Television, and Cartoons

The relationship between cartoons, movies, television, and PA levels in children is complex and multifaceted. Research indicates that media consumption, particularly television, is often associated with increased sedentary behavior and decreased PA among children [123,124]. A study [125] that analyzed 120 episodes of teen television shows found that PA was infrequently portrayed, accounting for only 3.2% of total viewing time. The activities shown were mostly vigorous and recreational, such as dancing and running, and were often motivated by enjoyment. Similarly, in cartoons, various authors [126] found that some television cartoons tend to depict sedentary behaviors more frequently than physical activities, which may influence children’s perceptions and behaviors regarding PA. Another content analysis of popular teen television shows found that while both male and female characters participated in physical activities, men were more often depicted in competitive sports [127]. However, there are instances where media can positively influence PA levels, depending on the content and context. This nuanced relationship highlights the need for strategic interventions to balance media consumption with PA in children’s daily routines. For example, certain media content, such as sports-themed cartoons, can positively influence children’s interest in physical activities. For example, the anime *Attack No. 1* led to a significant increase in volleyball participation among girls in Germany [128]. Cartoons can also educate children about physical activities, as demonstrated by a study in which animated media improved students’ knowledge and attitudes toward PA [129]. PA experiences (PAEs) based on popular films can also lead children to engage in enjoyable physical activities, suggesting that media can be used creatively to promote PA [130].

#### 3.4.6. Advertising

Advertising has a significant impact on children’s PA levels, as demonstrated by several studies. Campaigns such as the VERB campaign (VERB: It’s what you do) promoted by the US Centers for Disease Control (CDC) have shown that targeted advertising can increase children’s awareness and participation in PA. The effectiveness of these campaigns is often influenced by factors such as age, baseline activity levels, and the medium used for advertising. The VERB campaign, aided by the media, was able to increase PA among children aged 9 to 13 by using commercial marketing methods to make PA seem fun and socially rewarding. After one year, 74% of children were aware of the campaign, and those who were more aware engaged in more physical activity sessions, especially among younger children, girls, and those in urban areas [131].

In Arkansas, paid radio ads also promoted PA among preteens, and 76.1% of those exposed to the ads reported that they were more likely to engage in PA. Younger preteens were more influenced by these ads than older preteens [132]. But in contrast, some authors [133,134] have warned of the danger of advertising (commercials) that show physical activities together with unhealthy food products, since they can influence children’s perception of the healthiness of foods. Studies show that children, especially younger ones, can develop more positive attitudes toward these foods when they are associated with physical activities, although this does not necessarily translate into an increase in PA.

Finally, it is worth highlighting the results of a systematic review [135] which found that, in general, digital media strategies have been identified as potential tools to increase PA levels among children, although further research is needed to confirm these findings due to the heterogeneity of exercise protocols.

#### 3.4.7. Other Considerations

The aim of this work was to analyze all possible contexts where children and young people can engage in PA, thereby increasing their daily levels of PA. Significant scientific support was found in all of them, which should encourage political, social, and educational authorities and families to establish programs that promote and dynamize PA in all settings, thus reducing the time of physical inactivity and avoiding a sedentary lifestyle.

There are also other studies [136] which have found that holistic interventions in schools can improve children’s physical literacy skills. It is important to highlight the relevance reflected in the scientific literature toward this last term, physical literacy, with a consensus being published which pointed out that physical literacy is our relationship with movement and PA throughout life [137].

On the other hand, some studies [138] have focused on the important correlation that may exist between PA levels and other areas of development such as cognitive function or academic performance. This last study found evidence suggesting that there are positive associations between PA, physical fitness, cognition, and academic performance, although in some cases it may be inconsistent. The authors now pose the challenge of knowing what type of PA is best, how much, how often, and when to do it.

Finally, it is important to note the findings of a recent paper [38] that outlined several principles linked to PA practice. First, to highlight that any amount of PA contributes to health benefits, i.e., there is no minimum PA threshold for which health benefits need to be obtained. Another principle that these authors highlighted is that periods of 10 min or more of PA are not required to count as higher PA. Any period of time will always add up. Pending further studies, new accelerometry data suggest that intermittent intense PA of just 1–2 min can also benefit health, such as that acquired by climbing stairs or carrying heavy shopping bags. This has given rise to a new concept of promoting “lifestyle physical activity”. Third, excessive sedentary behavior is detrimental to health if not accompanied by sufficient daily physical activity or if accumulated over long, uninterrupted periods. Fourth, incorporating endurance exercise is essential for optimal health and promoting successful aging. Fifth, a large body of emerging evidence indicates that physical activity strongly protects and promotes all aspects of brain health and can improve cognition even after a single bout of exercise.

## 4. Conclusions

The present study has fulfilled the objective of analyzing various contexts in which an increase in PA levels can be promoted among the child and youth population. Through a review of the scientific literature, we have shown that school, extracurricular, family, and socio-community contexts offer key opportunities for the development of effective programs and strategies. Each of these contexts has specific characteristics that make them ideal spaces to implement interventions that promote regular PA, which is essential to combat the increasing sedentary lifestyle at these stages of life.

In the school context, it was found that PE classes and active recreation programs have a positive impact on increasing PA levels. However, the limited duration of PE classes in many educational systems demands a greater implementation of complementary programs, such as active recess and motor breaks, that optimize the time available in educational centers to promote movement. Or alternatively, it is necessary to increase the hours of PE to meet the minimum requirements recommended by the WHO.

On the other hand, the extracurricular context offers ample possibilities through organized sports activities and free play in the street. These activities are essential to complement the unstructured time outside the classroom, where children can develop motor and social skills. However, there are variations in the effectiveness of these programs according to socioeconomic and cultural contexts, which raises the need to adapt interventions to the characteristics of each community.

The family context also plays a crucial role in promoting PA. The involvement of parents and caregivers as role models and active participants is essential. The review of studies has shown that active families and home-centered interventions, such as the use of active toys or video games with movement, can significantly influence children’s activity levels, although challenges related to time and resource limitations persist.

Finally, in the socio-community context, the availability of adequate infrastructure, such as parks and green areas, emerges as a determining factor in promoting PA. In addition, it was observed that the influence of social networks and influential people can have a positive impact on the motivation of young people to adopt more active lifestyle habits.

### Limitations of the Study and Possible Future Lines of Work

The main limitation of this work is the possible bias that may exist in the selection of articles. Despite the fact that the researchers have carried out an exhaustive search and selection of articles, incorporating the most relevant articles in this work and following the established inclusion criteria, there may always be some relevant work that was not referenced. Bias in literature reviews is always present. In the development of this research, an attempt was made to minimize it, and the search process especially was correctly detailed to inform readers, as several researchers participated in the search and selection process.

In the future, it will be essential to develop comprehensive strategies that integrate multiple contexts and focus efforts on the creation of public policies that favor accessibility to spaces for PA, regardless of the socioeconomic context. Likewise, the long-term effects of interventions in different contexts must continue to be evaluated, especially those that involve the use of emerging technologies, such as portable devices and active video games, which offer novel opportunities for participation.

Furthermore, it is important to study how these interventions can influence other areas of development, such as cognition, socio-emotional aspects, and academic performance, in order to offer a more holistic approach to promoting PA in the child and adolescent population. Finally, future research should focus on the cultural adaptation of the programs to ensure their effectiveness in diverse communities and explore the impact of new technologies in the educational and family environment.

## Data Availability

No new data were created or analyzed in this study. Data sharing is not applicable to this article.
